# Dietary American Cockroach (*Periplaneta americana*) Residue in Juvenile Black Carp (*Mylopharyngodon piceus*): Effects on Growth, Antioxidant Status, Metabolism, and Whole-Body Amino Acid Profile

**DOI:** 10.3390/ani16152398

**Published:** 2026-08-03

**Authors:** Wei Pu, Youjiao Wu, Chenggui Zhang, Tingting Hu, Zhuanxing Shao, Jingjing Gao, Liyun Pu, Weile Chu, Xiaowen Long

**Affiliations:** 1 College of Agriculture and Biological Science, Dali University, Dali 671003, China; 20221109020112@dali.edu.cn (W.P.); 20221109020157@dali.edu.cn (Y.W.); htt@stu.dali.edu.cn (T.H.); szx@stu.dali.edu.cn (Z.S.); gjj@stu.dali.edu.cn (J.G.); ply@stu.dali.edu.cn (L.P.); cwl@stu.dali.edu.cn (W.C.); 2 National Observation and Research Station of Erhai Lake Ecosystem in Yunnan, Dali University, Dali 671003, China; 3 Co-Innovation Center for Cangshan Mountain and Erhai Lake Integrated Protection and Green Development of Yunnan Province, Dali University, Dali 671003, China; 4 Yunnan Provincial Key Laboratory of Entomological Biopharmaceutical R&D, Dali University, Dali 671000, China; chenggui_zcg@hotmail.com; 5 National-Local Joint Engineering Research Center of Entomoceutics, Dali 671000, China

**Keywords:** *Periplaneta americana* residue, *Mylopharyngodon piceus*, insect-derived feed ingredient, antioxidant status, metabolism, whole-body amino acid profile

## Abstract

This study evaluated the efficacy of post-extraction American cockroach (*Periplaneta americana*) residue (PAR) as a dietary feed ingredient on the growth performance, antioxidant defense, and whole-body amino acid profile of juvenile black carp (*Mylopharyngodon piceus*). A total of 300 juvenile black carp (initial weight 17.19 ± 0.20 g) were fed five isonitrogenous and isolipidic diets for 64 days. PAR was supplemented at inclusion levels of 0% (control), 5%, 10%, 15%, and 20% (corresponding to progressive reductions in dietary fishmeal). The results showed that fish fed the diet with 5% PAR inclusion maintained growth performance and feed conversion efficiency comparable to those of the control group (*p* > 0.05). Thus, 5% PAR did not significantly improve growth performance, but it did not impair growth under the present experimental conditions. Moreover, 5% PAR supplementation significantly increased hepatic catalase activity and was associated with numerically higher values of several other antioxidant- and immune-related indices. PAR inclusion also increased whole-body glycine deposition, whereas high inclusion levels reduced whole-body methionine content and crude protein deposition. Dietary PAR inclusion at higher levels, especially 20%, was associated with a possible reduction in feed utilization, altered organ indices, altered serum glucose levels, suppressed hepatic transaminase activities, and changes in digestive enzyme activities. However, these responses should be interpreted as the combined outcome of ingredient substitution, amino acid imbalance, altered lipid source, possible essential fatty acid changes, chitin/fiber-like fractions, and metabolic burden, rather than as effects of chitin alone. In conclusion, 5% PAR may be a suitable practical inclusion level for juvenile black carp under the present experimental conditions, whereas excessive inclusion should be used with caution and may require amino acid balancing, improved processing, and further evaluation of dietary gross energy, fatty acid profile, and chitin content.

## Simple Summary

Fishmeal is an important but increasingly limited ingredient in aquaculture feeds, so alternative and sustainable feed resources are needed. American cockroach residue is a by-product generated after the extraction of useful compounds from American cockroaches, and it still contains nutrients and functional substances. This study evaluated whether this residue could be used in diets for juvenile black carp. The results showed that 5% American cockroach residue maintained growth and feed utilization at levels comparable to the control diet, while also improving selected liver antioxidant responses. However, higher inclusion levels were associated with less favorable feed utilization, changes in metabolism, and alterations in whole-body amino acid composition. Because increasing American cockroach residue also changed the overall ingredient composition of the diets, including reductions in fishmeal, fish oil, and soybean oil, these high-inclusion effects should not be attributed to chitin alone. These findings suggest that a moderate level of American cockroach residue may be useful as a sustainable feed ingredient for black carp, but excessive inclusion should be avoided or further optimized through better diet formulation.

## 1. Introduction

With the rapid transformation of global aquaculture toward intensification, the demand for high-quality and sustainable diet ingredients has become increasingly urgent. Historically, fishmeal (FM) has maintained its dominant position in aquafeeds for carnivorous fish due to its high crude protein content, balanced essential amino acid profile, and excellent palatability [1,2]. However, heavily impacted by the decline of marine fishery resources and extreme climate events, the stability of global FM supply has significantly declined [3,4]. This escalating contradiction has prompted animal nutritionists to shift their strategic focus: rather than solely screening widely available macronutrient replacements [5,6], there is an urgent need to develop novel, green feed ingredients that provide both nutritional support and unique physiological regulatory functions. Consequently, insect-derived ingredients are demonstrating excellent prospects not only due to their high feed conversion efficiency and eco-friendly advantages but also because they serve as a valuable natural reservoir of bioactive compounds [7,8].

Among various promising resource insects, the American cockroach (*Periplaneta americana*) contains various bioactive substances, such as antimicrobial peptides, immunopolysaccharides, polyols, and specific alkaloids [9]. Modern biopharmaceutical research confirms that these components have been reported to show physiological regulatory potential in modulating glucose and lipid metabolism, resisting oxidative stress, and activating non-specific immune defense barriers [9,10]. *Periplaneta americana* residue (PAR) is the primary solid by-product obtained after intensive solvent extraction in the pharmaceutical industry [11]. Notably, despite undergoing extraction, PAR retains a certain amount of insect-derived protein and residual bioactive components with potential immunostimulatory effects. Utilizing PAR as a functional ingredient in the aquafeed industry offers a dual benefit: providing potential immunomodulatory properties to the formulated diets while achieving the green recycling of pharmaceutical solid wastes.

Previous systematic explorations have confirmed the application potential of *P. americana* derivatives in various economic fish species. In cold-water carnivorous rainbow trout (*Oncorhynchus mykiss*), appropriate dietary inclusion of *P. americana* powder or residue significantly influenced growth performance, antioxidant capacity, and non-specific immune responses [12,13,14,15]. Similarly, moderate dietary introduction of PAR exerts regulatory effects on circulatory immune parameters and hepatic antioxidant barriers in omnivorous Nile tilapia (*Oreochromis niloticus*) and common carp (*Cyprinus carpio*) [11,16]. Therefore, the novelty of the present study does not lie simply in testing *P. americana*-derived material in another fish species, but in determining the species-specific tolerance threshold and physiological response pattern of juvenile black carp under graded PAR inclusion. In addition, insect-derived feed ingredients may differ markedly in their protein quality, amino acid profile, lipid and fatty acid composition, chitin/fiber-like fractions, and residual bioactive compounds [7,8,17,18]. Thus, their effects should be evaluated as the integrated outcome of overall ingredient substitution rather than being attributed to a single component.

Black carp (*Mylopharyngodon piceus*), one of the highly renowned carnivorous freshwater “Four Major Chinese Carps,” occupies a crucial strategic position in China’s aquaculture industry due to its rapid growth rate, large commercial size, and firm, delicious meat quality [19], with annual production surpassing 800,000 tons in 2023 [20]. As a benthic and molluscivorous/carnivorous fish naturally adapted to crushing and consuming hard-shelled organisms, black carp may possess digestive and physiological responses to insect-derived residues that differ from those of rainbow trout, Nile tilapia, and common carp [21]. However, whether this feeding habit confers greater tolerance to PAR remains unverified and requires experimental evaluation. Although there have been several reports on utilizing traditional plant proteins in black carp commercial diets [22,23], the systematic evaluation of novel PAR as a functional additive—specifically regarding its optimal inclusion threshold, its effects on amino acid deposition and selected quality-related traits, and its associated physiological and metabolic responses—has not been systematically investigated.

Consequently, under the conventional principle of isonitrogenous and isolipidic feed formulation, this study conducted a 64-day gradient dietary inclusion trial to determine the suitable inclusion level of PAR in the diets of juvenile black carp. PAR was supplemented at actual dietary inclusion levels of 0%, 5%, 10%, 15%, and 20%. The 0% PAR diet served as the control. The 5% PAR level was selected as a low practical inclusion level for partial fishmeal replacement and functional evaluation based on previous studies using *P. americana*-derived materials and other insect-derived feed ingredients in fish [11,12,13,14,15,16,24,25]. The higher inclusion levels of 10–20% were designed to evaluate the tolerance threshold and potential adverse effects of graded PAR inclusion in juvenile black carp [11,16,24,25]. This study aimed to systematically evaluate the effects of dietary PAR inclusion on growth performance, tissue digestive enzyme activities, antioxidant and immune-related responses, metabolic status, and whole-body amino acid profile. We hypothesized that low-level PAR inclusion would maintain growth performance and improve selected antioxidant responses, whereas excessive inclusion could disturb feed utilization and metabolic homeostasis because of combined changes in dietary ingredient composition, amino acid balance, lipid source, and indigestible fractions.

## 2. Materials and Methods

### 2.1. Ethics Statement

This study was conducted at the Agronomy Experimental Center of the College of Agriculture and Biological Science, Dali University, Yunnan Province, People’s Republic of China. All experimental protocols and procedures were approved by the Animal Welfare and Ethical Committee of Dali University (ethics approval code: 2020-PZ-46). Throughout the experimental period, fish were maintained in well-aerated water conditions. Prior to sampling, specimens were anesthetized using eugenol to minimize stress and discomfort. All tissue collection and visceral extraction procedures were performed in strict accordance with the Guidelines for the Care and Use of Laboratory Animals in China, ensuring adherence to established ethical standards for animal experimentation.

### 2.2. Experimental Diet Preparation

Five isonitrogenous and isolipidic experimental diets were formulated. Fishmeal (FM) and soybean meal served as the primary protein sources, while fish oil and soybean oil were utilized as the main lipid sources. The PAR used in this study was the solid by-product generated after extraction of bioactive compounds from dried *Periplaneta americana* during pharmaceutical processing. To evaluate the efficacy of the functional additive, *Periplaneta americana* residue (PAR) was supplemented at inclusion levels of 0% (Diet 1, control), 5% (Diet 2), 10% (Diet 3), 15% (Diet 4), and 20% (Diet 5) of the total diet. The 5% level was selected as a low practical inclusion level for partial fishmeal replacement and functional evaluation based on previous studies using *P. americana*-derived materials and other insect-derived feed ingredients in fish [11,12,13,14,15,16,24,25]. The 10%, 15%, and 20% levels were designed to evaluate the response of juvenile black carp to graded and high-level PAR inclusion [11,16,24,25].

To maintain the isonitrogenous and isolipidic formulation targets, the progressive inclusion of PAR was balanced mainly by corresponding reductions in fishmeal and supplemental lipid sources. Because PAR contained less crude protein but more crude lipid than fishmeal, fish oil and soybean oil were progressively reduced as dietary PAR increased. Therefore, although the diets were formulated to be isonitrogenous and isolipidic based on proximate composition, the fatty acid profile and gross energy level may have differed among diets. Dietary gross energy, fatty acid composition, and chitin content were not directly measured in the present study; this limitation was considered when interpreting the physiological responses to high PAR inclusion.

All raw pulverized ingredients were passed through a 60-mesh standard sieve and precisely weighed according to the proportions in Table 1. The ingredients were thoroughly mixed using a progressive homogeneous blending method. Subsequently, distilled water (30% of the total diet weight) was added, and the mixture was thoroughly blended. Sinking pellets with a diameter of 1.50 mm were produced using a laboratory pelletizer. The prepared diets were air-dried naturally at room temperature, sealed in self-sealing bags, and stored at −20 °C in the dark until feeding. The formulation and proximate composition of the experimental diets are presented in Table 1, and the measured proximate and amino acid compositions of the core ingredients and diets are shown in Table 2.

### 2.3. Experimental Fish and Husbandry Management

The aquaculture experiment was conducted at the Agronomy Experimental Center of the College of Agriculture and Biological Science, Dali University. Juvenile black carp (*Mylopharyngodon piceus*) were obtained from a certified commercial hatchery in Binchuan, Dali. Upon arrival, the fish were bathed and disinfected in a 3% salt solution for 5 min. A total of 300 healthy and uniform juveniles (mean initial body weight: 17.19 ± 0.20 g) were randomly distributed into 15 independent recirculating aquaculture system (RAS) tanks (0.6 m × 0.36 m × 0.72 m). The experiment comprised 5 treatment groups with 3 replicates per group and 20 fish per replicate. Before the formal trial, fish were acclimated to the experimental conditions and fed the control diet for 7 days. The formal feeding trial lasted for 64 days. Fish were hand-fed twice daily at 08:00 and 17:00 to apparent satiety (the daily feeding rate was approximately 3–7% of body weight, adjusted flexibly based on daily ingestion, growth trends, and water temperature). During the trial, a natural photoperiod was maintained. The water temperature was regulated at 27.0 ± 1.0 °C, dissolved oxygen was kept at ≥ 5.0 mg/L via continuous aeration, and the pH ranged from 7.0 to 7.5. Feces and uneaten feed at the bottom of the tanks were siphoned out daily, and approximately 1/3 of the water volume was exchanged depending on water quality to ensure a clean and stable environment.

### 2.4. Sample Collection

At the end of the 64-day feeding trial, all fish were fasted for 24 h. The total number and weight of surviving black carp in each tank were recorded to calculate survival rates and growth performance. Prior to sampling, fish were deeply anesthetized with a 30 mg/L eugenol solution. Five fish were randomly selected from each replicate tank to measure body length and weight for the calculation of the condition factor. Blood samples were collected from the caudal vein using 1.0-mL sterile syringes. The whole blood samples were allowed to stand at 4 °C for 4 h, and then centrifuged at 10,000× *g* for 10 min at 4 °C to separate the serum. The supernatant serum was immediately aliquoted and stored at −40 °C for subsequent biochemical and immunological assays. After blood collection, the fish were immediately dissected on ice to separate the visceral mass and liver tissue, which were precisely weighed to determine the viscerosomatic index (VSI) and hepatosomatic index (HSI). Portions of the liver and anterior intestine tissues were rinsed with ice-cold physiological saline and stored at −40 °C for the determination of tissue metabolism, digestive enzyme activities, and antioxidant capacity. The remaining whole-body fish and undissected samples were blotted dry, placed in self-sealing bags, and stored at −20 °C for proximate composition analysis.

### 2.5. Proximate Composition Analysis of Diets and Whole Fish

According to the AOAC procedure [26], the moisture content (method 930.15), crude protein (Kjeldahl method, nitrogen content (N) × 6.25) (method 968.06), and ash content (method 942.05) of fishmeal, PAR, experimental diets, and fish samples were analyzed. Briefly, dry matter was determined by drying samples overnight (12 h) in an oven at 105 °C until a constant weight was reached. Crude protein was determined using the Kjeldahl method with a conversion factor of 6.25. Ash content was determined by incineration at 550 °C for 5 h, and total lipids were determined according to the method described by Folch et al. [27]. The amino acid composition of the fishmeal, PAR, experimental diets, and fish samples was analyzed following the methods described by Blackburn [28]. Approximately 0.1 g of each sample was placed separately in 40 mL ampules with 8 mL of 6 mol·L^−1^ HCl. The ampules were vacuum-sealed, and the samples were hydrolyzed at 110 °C for 24 h. The hydrolysates were then dissolved in 50 mL of distilled water and centrifuged at 2659× *g*. The supernatants were filtered through a 0.22 μm filter (Millipore, Bedford, MA), and 1 mL of the filtered supernatant was retrieved and vacuum-dried at 50 °C to remove the initial HCl. Then, 2–5 mL of 0.02 mol·L^−1^ HCl was added, and 1 mL of the final sample was analyzed using an amino acid analyzer (Hitachi L-8900, Hitachi, Ltd., Tokyo, Japan).

### 2.6. Tissue Digestive Enzyme and Biochemical Metabolism Assays

Biochemical indicators in tissues and serum were measured using a T6 New Century UV-Vis spectrophotometer (Beijing Purkinje General Instrument Co., Ltd., Beijing, China) and commercial diagnostic kits (Nanjing Jiancheng Bioengineering Institute, Nanjing, China) strictly following the manufacturer’s protocols. In the intestinal and hepatic tissues, trypsin activity was determined via the UV-Vis spectrophotometric method, lipase activity was quantified colorimetrically, and α-amylase activity was measured based on the starch–iodine colorimetric method. Serum and hepatic metabolic indicators were determined using enzymatic assays, including serum blood urea nitrogen (BUN), total protein (TP), triglycerides (TG), glucose (GLU), total cholesterol (T-CHO), total free amino acids (TAA), and serum aspartate aminotransferase (AST) activity. Hepatic glycogen content, alanine aminotransferase (ALT) activity, and AST activity were also determined.

### 2.7. Antioxidant Capacity and Immunological Assays

The activities of alkaline phosphatase (AKP), acid phosphatase (ACP), catalase (CAT), total antioxidant capacity (T-AOC), and glutathione peroxidase (GSH-Px) in the liver and serum samples were determined using specific kits from the Nanjing Jiancheng Bioengineering Institute. Serum immunoglobulin M (IgM), complement C3 levels, and hepatic lysozyme (LZM) activity were quantified using the corresponding specific diagnostic kits from the same manufacturer.

### 2.8. Calculations of Growth Performance and Morphometric Indices

The indices were calculated using the following standard formulas [11,16,24,25]:

SR (%) = (N_t_/N_0_) × 100

WGR (%) = [(W_t_/N_t_ − W_0_/N_0_)/(W_0_/N_0_)] × 100

SGR (%/day) = [(ln (W_t_/N_t_) − ln (W_0_/N_0_))/t] × 100

FCR = F/(W_t_ + W_d_ − W_0_)

DFR (%) = [2F/(t×(W_0_ + W_t_ + W_d_))] × 100

CF (%) = (W/L^3^) × 100

VSI (%) = (W_v_/W) × 100

HSI (%) = (W_h_/W) × 100

where N_0_ and N_t_ are the initial and final number of fish per tank, respectively; W_0_ and W_t_ are the initial and final total body weight per tank (g), respectively; F is the total dry feed consumed during the trial (g); t is the experimental duration (days); W_d_ is the total body weight of fish that died during the feeding trial (g), which was included in the calculations of feed conversion ratio and daily feeding rate; W is the individual body weight of the sampled fish (g); L is the individual body length of the sampled fish (cm); W_h_ is the wet liver weight of the individual fish (g); and W_v_ is the wet visceral mass weight of the individual fish (g).

### 2.9. Statistical Analysis

All data are presented as mean ± standard error (mean ± SE). One-way analysis of variance (ANOVA) was performed using SPSS 25.0 statistical software. For growth performance and feed utilization variables, the replicate tank was used as the experimental unit. For biochemical, digestive enzyme, antioxidant, immune-related, and whole-body composition variables, values obtained from sampled fish within each tank were averaged before statistical analysis, and the tank-level mean was used as the experimental unit whenever applicable. Prior to analysis, data were subjected to Levene’s test for homogeneity of variance. If data met the assumptions of normal distribution and homogeneity of variance, Duncan’s multiple range test was used to evaluate the significance of differences among groups. If variances were heterogeneous but normality was satisfied, Welch’s ANOVA followed by the Games–Howell post-hoc test was used. If data did not meet the normality assumption after transformation, the Kruskal–Wallis H test followed by Dunn’s post-hoc test was applied. The significance level was set at *p* < 0.05. Principal component analysis (PCA) was conducted based on tank-level mean values of growth performance, metabolic parameters, antioxidant and immune indices, and amino acid-related variables. Spearman correlation analysis was performed to explore relationships between PAR inclusion level and physiological variables. PCA score plots and correlation heatmaps were generated using OriginPro 2021. Before PCA, all variables were standardized using Z-score transformation to minimize the influence of different units and scales.

## 3. Results

### 3.1. Growth Performance and Morphometric Indices

As shown in Table 3, the graded inclusion levels of PAR had no statistically significant effects on the survival rate, final body weight, weight gain rate, specific growth rate, and feed conversion ratio of juvenile black carp (*p* > 0.05). Although the final body weight, weight gain rate, and specific growth rate were numerically highest in Diet 2, these differences were not statistically significant. Therefore, 5% PAR inclusion should be interpreted as maintaining growth performance comparable to the control group, rather than producing superior growth performance. The feed conversion ratio showed a numerical increasing tendency with increasing PAR levels, suggesting a possible reduction in feed utilization efficiency in the high-inclusion groups. Significant differences were observed in the daily feeding rate among the groups (*p* < 0.05), with Diet 2 exhibiting a significantly lower daily feeding rate than Diets 3 and 4.

The viscerosomatic index in Diet 5 was significantly higher than that in all other groups (*p* < 0.05). The hepatosomatic index in Diet 5 was significantly higher than that in Diets 1 and 4, but did not differ significantly from that in Diets 2 and 3 (*p* > 0.05). No significant differences in either the viscerosomatic index or hepatosomatic index were detected among Diets 1–4 (*p* > 0.05). The condition factor fluctuated among the groups, reaching its highest value in Diet 3 and its lowest value in Diet 5. The condition factor in Diet 5 was significantly lower than that in Diet 3 (*p* < 0.05), whereas no significant differences were observed among the remaining treatments.

### 3.2. Whole-Body Composition and Amino Acid Profile

The whole-body proximate composition is presented in Table 4. Dietary PAR inclusion had no significant impact on the crude lipid and ash contents of the whole fish (*p* > 0.05). However, whole-body crude protein content differed significantly among treatments. It was significantly lower in Diets 4 and 5 than in the control group (*p* < 0.05), whereas Diets 2 and 3 did not differ significantly from the control group (*p* > 0.05).

Regarding the whole-body amino acid profile (Table 4), the contents of most individual amino acids and the total amino acids remained stable across all groups (*p* > 0.05), with the exception of methionine and glycine. In the essential amino acid sequence, methionine content progressively decreased with increasing PAR levels, and was significantly lower in Diet 5 compared to Diets 1 and 2 (*p* < 0.05). Conversely, in the non-essential amino acid sequence, glycine content demonstrated a significant positive dose-dependent accumulation, with the glycine level in Diet 5 significantly surpassing those in Diets 1 and 2 (*p* < 0.05). The ratio of essential to total amino acids in the 20% PAR inclusion group (Diet 5) was significantly lower than that in Diet 2 (*p* < 0.05).

### 3.3. Tissue Digestive Enzyme Activities

**Figure 1:**
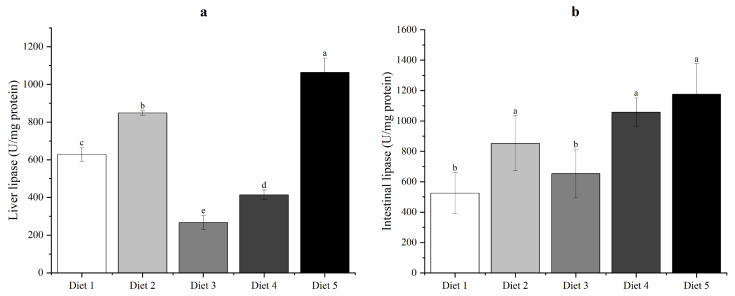
Lipase activity in the liver (**a**) and intestine (**b**) of juvenile *Mylopharyngodon piceus*. Data are presented as mean ± SE (*n* = 3). All data in this figure met the assumptions of normal distribution and homogeneity of variance. Bars with different letters indicate significant differences according to Duncan’s test (*p* < 0.05).

**Figure 2:**
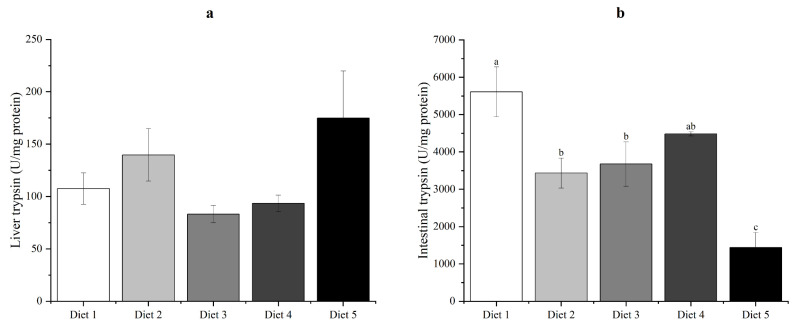
Trypsin activity in the liver (**a**) and intestine (**b**) of juvenile *Mylopharyngodon piceus*. Data are presented as mean ± SE (*n* = 3). All data in this figure met the assumptions of normal distribution and homogeneity of variance. Bars with different letters indicate significant differences according to Duncan’s test (*p* < 0.05).

Tissue digestive enzyme activities exhibited significant tissue-specific heterogeneous responses (Figure 1 and Figure 2). In the liver, α-amylase and trypsin activities remained stable across all treatments (*p* > 0.05). However, hepatic lipase activity in Diet 5 was significantly higher than that in all other groups (*p* < 0.05). Analysis of intestinal digestive function revealed that intestinal trypsin activity was highest in the control group and was significantly higher than that in Diets 2, 3, and 5 (*p* < 0.05). Concurrently, intestinal lipase activity in Diet 5 was significantly higher than that in Diets 1 and 3 (*p* < 0.05).

### 3.4. Serum and Hepatic Metabolic Biochemical Indices

As summarized in Table 5, hepatic transaminase activities were highly sensitive to high levels of PAR intake. Hepatic alanine aminotransferase activity in Diet 5 was significantly lower than that in the control and Diet 4 (*p* < 0.05). Similarly, aspartate aminotransferase activity exhibited a progressive decline, dropping to its lowest point in Diet 5, which was significantly lower than the control level (*p* < 0.05).

Regarding serum metabolism, serum blood urea nitrogen content was significantly higher in Diet 3 than in all other groups (*p* < 0.05). No significant differences were found in serum total free amino acid contents and aspartate aminotransferase activities among the groups (*p* > 0.05). For lipid metabolism, serum triglycerides and total cholesterol did not differ significantly among treatments (*p* > 0.05). In terms of carbohydrate metabolism, hepatic glycogen content did not differ significantly among the groups. Nevertheless, serum glucose levels were significantly higher in Diets 3 and 4 than in Diet 2 (*p* < 0.05), whereas no significant differences were detected between the control group and the other treatments (*p* > 0.05).

### 3.5. Antioxidant Capacity and Immunity Indices

As shown in the antioxidant defense and immune response parameters summarized in Table 6, low-level dietary inclusion was associated with higher selected hepatic antioxidant and immune-related enzyme activities. In the hepatic tissue, the activities of acid phosphatase, catalase, total antioxidant capacity, and glutathione peroxidase were numerically highest in Diet 2 (5% PAR inclusion). Notably, CAT activity in Diet 2 was significantly higher than that in the other groups (*p* < 0.05). Hepatic lysozyme activity, however, did not differ significantly among treatments.

Analysis of circulating serum indicators revealed that PAR inclusion did not cause marked changes in the activities or concentrations of alkaline phosphatase, acid phosphatase, catalase, total antioxidant capacity, complement C3, and specific antibody IgM in the circulatory system (*p* > 0.05). Conversely, serum glutathione peroxidase activity was significantly higher in Diet 4 than in the other groups (*p* < 0.05). When the inclusion level reached 20% (Diet 5), several antioxidant and immune-related parameters in the liver and serum were lower than those in the control group.

### 3.6. Principal Component Analysis and Correlation Analysis

**Figure 3:**
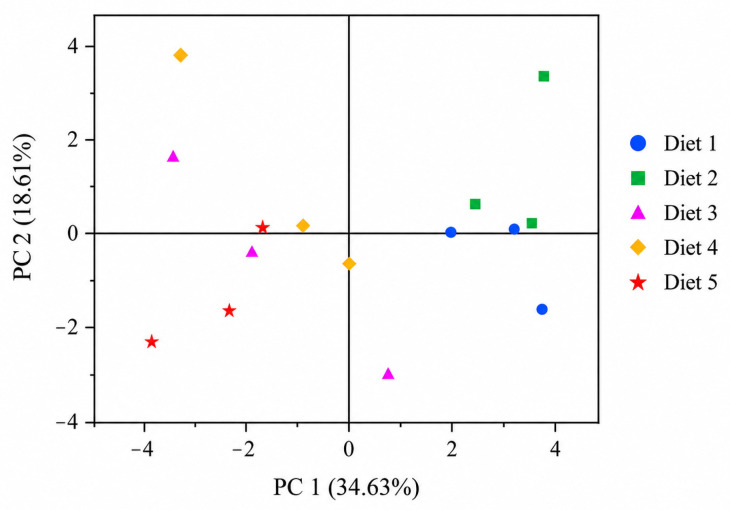
Principal component analysis (PCA) score plot based on growth performance, metabolic parameters, antioxidant and immune indices, and amino acid-related variables of juvenile black carp fed diets containing different levels of *Periplaneta americana* residue. Each point represents one replicate tank. Diet 1, Diet 2, Diet 3, Diet 4, and Diet 5 correspond to diets supplemented with 0%, 5%, 10%, 15%, and 20% *P. americana* residue, respectively. PC1 and PC2 explained 34.63% and 18.61% of the total variance, respectively.

To provide an integrated view of the responses of juvenile black carp to dietary *Periplaneta* americana residue (PAR), PCA was performed based on growth performance, metabolic parameters, antioxidant and immune indices, and amino acid-related variables. As shown in Figure 3, PC1 and PC2 explained 34.63% and 18.61% of the total variance, respectively. The PCA score plot showed that the replicate tanks from different dietary treatments displayed a certain degree of separation, indicating that graded PAR inclusion affected the overall physiological profile of juvenile black carp. In particular, the distribution of Diet 2 was closer to that of the control group than that of the higher inclusion groups, suggesting that 5% PAR inclusion maintained a physiological response pattern relatively similar to the control.

**Figure 4:**
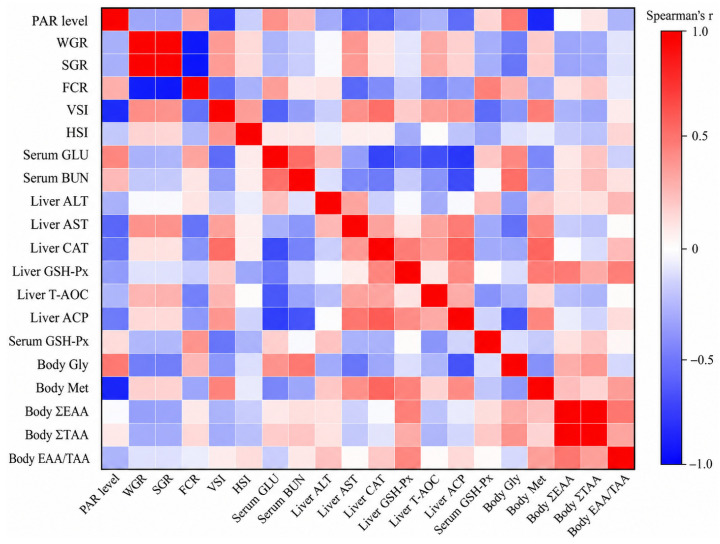
Spearman correlation heatmap showing relationships among dietary *Periplaneta americana* residue level, growth performance, metabolic parameters, antioxidant and immune indices, and whole-body amino acid composition of juvenile black carp. Red indicates positive correlations, whereas blue indicates negative correlations. The color intensity represents the strength of Spearman’s correlation coefficient.

Spearman correlation analysis was used to further examine relationships among PAR level, growth performance, metabolic status, antioxidant and immune responses, and whole-body amino acid composition (Figure 4). The correlation heatmap showed that PAR level was negatively correlated with WGR and SGR, but positively correlated with FCR and body glycine. Growth-related variables were positively associated with several antioxidant-related indices, whereas FCR, VSI, and HSI tended to be negatively associated with growth and selected antioxidant parameters. In addition, body methionine showed a negative association with PAR level. These results indicate that the effects of dietary PAR were closely related to changes in growth performance, metabolic status, antioxidant response, and amino acid deposition.

## 4. Discussion

The present study demonstrated that the inclusion of 5% *Periplaneta americana* residue (PAR) in the diet resulted in apparent growth performance and feed conversion efficiency equivalent to those of the control group. Because final body weight, weight gain rate, specific growth rate, and feed conversion ratio did not differ significantly between Diet 2 and the control group, the low-level PAR diet should be interpreted as growth-maintaining rather than growth-promoting. This growth-maintaining effect aligns with systematic findings in other economically important aquaculture species, indicating that moderate dietary inclusion of insect-derived ingredients or *P. americana*-derived products does not compromise, and occasionally enhances, growth performance [11,12,13,14,15,16,24,25,29,30,31]. For instance, similar growth-sustaining effects have been documented in juvenile rainbow trout (*Oncorhynchus mykiss*) fed PAR or black soldier fly (*Hermetia illucens*) meal [13,25], Nile tilapia (*Oreochromis niloticus*) fed PAR [11,31], golden pompano (*Trachinotus ovatus*) [29], and yellow catfish (*Pelteobagrus fulvidraco*) [30]. The equivalent growth output achieved through this low-level functional inclusion may be associated with the insect-derived protein retained in PAR, alongside small molecular active peptides and polysaccharide fragments that possess potential bioactive properties [7,9,10]. However, this interpretation should remain cautious because the present study did not directly characterize the bioactive components, chitin content, fatty acid profile, or gross energy of PAR and the experimental diets.

However, when PAR inclusion exceeded 10%, the weight gain rate showed a decreasing numerical trend and FCR increased numerically. These high-inclusion responses should not be attributed solely to chitin. In the present diet formulation, increasing PAR inclusion was accompanied by progressive reductions in fishmeal, fish oil, and soybean oil (Table 1). Therefore, the adverse responses observed at high PAR levels may reflect the combined effects of overall ingredient substitution, reduced dietary methionine supply, altered lipid source, possible changes in essential fatty acid availability, chitin/fiber-like fractions, and reduced nutrient utilization [7,8,17,18,31]. In particular, the measured amino acid composition showed a progressive reduction in dietary methionine with increasing PAR inclusion, and whole-body methionine was significantly reduced in the 20% PAR group (Table 2 and Table 4). This suggests that amino acid imbalance, especially methionine limitation, may have contributed to the reduced protein deposition and a possible reduction in feed utilization observed at higher PAR inclusion levels [32,33].

The exoskeleton of *P. americana* may contain chitin and other fiber-like components, which could affect digestive enzyme access to dietary nutrients [17,18]. Nevertheless, chitin content was not directly quantified in the present study. Therefore, chitin/fiber-like fractions should be regarded only as a possible contributing factor, not as a confirmed causal mechanism. Similarly, although black carp is a benthic and molluscivorous/carnivorous fish adapted to consuming hard-shelled organisms [21], the present data do not directly demonstrate enhanced chitin digestion or greater physiological tolerance to dietary chitin in this species. Future studies should measure dietary chitin content, endogenous chitinase activity, apparent nutrient digestibility, and intestinal histology to clarify this possibility.

The viscerosomatic index (VSI) and hepatosomatic index (HSI) are commonly used as indicators of organ development, nutrient partitioning, and physiological load in fish. In this study, the VSI and HSI were significantly increased in the 20% PAR group. This result may reflect altered nutrient deposition, changes in energy allocation, or increased metabolic burden under high PAR inclusion. However, because liver histology, lipid deposition, and pathological indicators were not examined, the increased VSI and HSI should not be directly interpreted as organ damage or pathological enlargement. Similar changes in organ indices have been reported in some fish fed high levels of insect-derived ingredients or alternative marine ingredients [34,35]. Nevertheless, in the present study, the high PAR diet differed from the control diet not only in PAR content but also in fishmeal, fish oil, soybean oil, amino acid profile, and probably fatty acid composition. Thus, the altered organ indices in the 20% PAR group should be interpreted as an integrated response to high-level ingredient substitution rather than as a specific effect of chitin or PAR alone.

Analysis of tissue nutrient deposition revealed that whole-body crude protein content declined in the high-PAR inclusion groups. This result should be interpreted in relation to the altered dietary amino acid profile rather than as direct evidence of impaired muscle synthesis. The measured amino acid composition of the diets showed that dietary methionine decreased progressively with increasing PAR inclusion, while whole-body methionine content also declined in the 20% PAR group. Methionine is an essential amino acid involved in protein synthesis, methyl-group metabolism, and growth regulation in aquatic animals [31,32]. Therefore, the reduction in dietary and whole-body methionine may have contributed to the lower whole-body crude protein deposition observed at high PAR inclusion levels. However, protein turnover, muscle histology, and amino acid metabolic flux were not measured in the present study; therefore, the mechanistic link between methionine limitation and protein deposition requires further verification.

Conversely, a notable observation of this study is the dose-dependent positive accumulation of glycine in the fish body induced by PAR inclusion. This targeted accumulation is likely driven by the inherently high glycine content in the PAR profile itself (4.10% dry matter, as shown in Table 2), which provides an abundant direct substrate for muscle deposition. In addition, PAR-derived nutrients or bioactive compounds may have influenced amino acid utilization, but the underlying metabolic pathways were not examined in the present study. Glycine is a taste-active amino acid that mainly contributes to sweet taste and may influence the overall non-volatile flavor profile of seafood and aquatic products [36]. Therefore, increased glycine deposition may indicate a potential effect of PAR on selected amino acid-related quality traits. However, because sensory evaluation, free amino acid composition of edible muscle, and flesh quality indicators were not systematically assessed, the present data are insufficient to conclude that PAR improves flesh quality. Future studies should evaluate muscle-free amino acids, sensory attributes, texture, and flavor-related metabolites to determine whether PAR has practical value for quality improvement.

Digestive enzyme activities are important indicators of the hydrolysis and utilization of dietary macronutrients. In this study, intestinal trypsin activity decreased with increasing PAR inclusion and reached the lowest level in the 20% PAR group, suggesting that high PAR inclusion may have affected protein digestive capacity or digestive enzyme regulation. This response may be related to the combined effects of ingredient substitution, amino acid imbalance, chitin/fiber-like fractions, and altered nutrient accessibility rather than to chitin alone [17,18]. At the same time, hepatic and intestinal lipase activities increased in the 20% PAR group. This increase should be interpreted cautiously. It may represent a response to altered dietary lipid source or lipid composition, a compensatory digestive–metabolic adjustment, or a sign of diet-induced metabolic stress. Because lipidomics, fatty acid profiles, bile acid metabolism, and liver or intestinal histology were not examined, these possibilities cannot be distinguished in the present study. Therefore, the digestive enzyme results support the view that high PAR inclusion altered digestive and metabolic responses, but they do not allow a definitive mechanistic conclusion.

Regarding biochemical metabolism, hepatic ALT and AST activities were reduced in the high-level PAR inclusion groups, suggesting that high PAR inclusion may have affected hepatic amino acid metabolism and transamination processes. Transaminase kinetics are closely associated with the rate of endogenous amino acid transamination [37]. However, because hepatic free amino acid pools, protein turnover, and related metabolic pathways were not directly measured, the reduced transaminase activities should not be interpreted as direct evidence that hepatocytes drastically slowed down protein synthesis or metabolic flux. In addition, serum glucose levels were increased in the higher PAR inclusion groups, suggesting that excessive PAR inclusion may disturb carbohydrate metabolic homeostasis to some extent. In fish, blood glucose is commonly used as a secondary physiological indicator of stress responses and is closely associated with cortisol release, catecholamine-mediated energy mobilization, and activation of the hypothalamic–pituitary–interrenal axis [38,39,40]. Therefore, the glucose response observed in the present study may indicate altered carbohydrate metabolism, but it may also reflect possible diet-induced metabolic stress rather than a simple adaptive energy response.

Because hepatic glycogen content did not differ significantly among treatments, the increased serum glucose may not be fully explained by glycogen mobilization alone. Fish glucose homeostasis is regulated by multiple nutritional and endocrine pathways, including hepatic glycogen metabolism, gluconeogenesis, glycolysis, insulin/glucagon signaling, and stress-related corticosteroid responses [41,42]. It is also possible that part of the circulating glucose was derived from gluconeogenesis using glucogenic amino acids or glycerol released during triglyceride mobilization [41,42]. However, insulin, glucagon, cortisol, hepatic gluconeogenic enzymes, glucose transporters, and related metabolic signaling pathways were not measured in the present study. Therefore, the endocrine and molecular mechanisms underlying the serum glucose response cannot be confirmed. Future studies should include glucose-regulatory hormones, cortisol, hepatic glycogen metabolism, gluconeogenesis-related genes or enzymes, glycerol, and glucogenic amino acid flux to determine whether high PAR inclusion induces carbohydrate metabolic imbalance or physiological stress [38,39,40,41,42].

Aquatic animals rely on endogenous antioxidant defense systems, including enzymatic antioxidants such as CAT and GSH-Px, to cope with reactive oxygen species and oxidative challenges under intensive aquaculture conditions [43]. In the present study, 5% PAR inclusion significantly increased hepatic CAT activity and was associated with numerically higher hepatic GSH-Px, T-AOC, and ACP activities. This response may be partly associated with bioactive compounds retained in *P. americana*-derived materials, such as peptides, polysaccharide-like fractions, and other antioxidant-related components [9,10,44,45,46]. Similar improvements in antioxidant or non-specific immune-related responses have also been reported in fish fed *P. americana*-derived products or other insect-derived feed ingredients [11,12,13,14,15,16,24,25,29,30]. These results suggest that low-level PAR inclusion may support hepatic antioxidant defense and certain non-specific immune-related responses. However, because inflammatory markers, pathogen challenge tests, and disease-resistance assays were not included, the present data are insufficient to conclude that PAR improves disease resistance. This potential should be further evaluated in future studies.

When PAR inclusion exceeded the suitable range, several antioxidant and immune-related parameters tended to decline, particularly in the 20% PAR group. The original interpretation of a typical “hormesis” or toxic inhibition effect should be weakened. The present results more conservatively indicate that high PAR inclusion may disturb antioxidant and immune-related homeostasis. This disturbance may be related to the combined effects of dietary formulation changes and nutrient utilization, including amino acid imbalance, altered lipid source, possible essential fatty acid differences, chitin/fiber-like fractions, and increased metabolic burden [7,8,17,18,31]. Therefore, the adverse responses observed at high PAR inclusion levels should be interpreted as the combined effects of dietary composition, nutrient utilization, metabolic status, and antioxidant capacity, rather than as evidence of a single direct toxic mechanism.

The PCA and correlation analyses further supported the dose-dependent physiological responses observed in the present study. The PCA score plot showed that the 5% PAR inclusion group was relatively close to the control group, whereas higher inclusion groups showed greater dispersion, suggesting that excessive PAR inclusion altered the overall physiological status of juvenile black carp. Moreover, the correlation heatmap revealed that PAR level was negatively associated with growth-related indicators and positively associated with FCR and body glycine, while body methionine showed a negative association with PAR level. These relationships suggest that the biological effects of PAR are not determined by a single indicator, but by the combined responses of growth performance, feed utilization, metabolism, antioxidant defense, and amino acid-related variables. However, PCA and correlation analyses are exploratory and cannot establish causal relationships. Therefore, correlations between PAR level, growth performance, metabolic indicators, antioxidant parameters, and amino acid deposition should be interpreted as hypothesis-generating evidence rather than mechanistic proof.

Several limitations should be acknowledged. First, although the diets were formulated to be isonitrogenous and isolipidic, dietary gross energy, fatty acid composition, and chitin content were not directly measured. Therefore, the effects of high PAR inclusion cannot be separated from changes in lipid source, possible essential fatty acid profile, energy supply, and chitin/fiber-like fractions. Second, apparent digestibility, chitinase activity, bile acid metabolism, lipidomics, and liver or intestinal histology were not examined, limiting the mechanistic interpretation of digestive enzyme and organ-index responses. Third, insulin, glucagon, cortisol, and glucose-regulatory signaling pathways were not measured; therefore, the mechanism underlying elevated serum glucose remains unclear. Finally, lower inclusion levels below 5% were not tested. Future studies should include 1–5% gradients, direct chitin and fatty acid profiling, digestibility measurements, amino acid balancing, and improved processing of PAR to determine the minimum effective dose and maximum practical inclusion level in black carp diets.

## 5. Conclusions

In summary, the effects of dietary PAR in juvenile black carp were dose-dependent. Under the present experimental conditions, 5% PAR inclusion maintained growth performance comparable to the control and improved selected hepatic antioxidant parameters, while higher inclusion levels, especially 20%, were associated with less favorable feed utilization, altered organ indices, altered metabolic parameters, and changes in amino acid deposition. However, these responses should not be attributed solely to chitin because high PAR inclusion was accompanied by changes in fishmeal level, lipid source, amino acid profile, and possibly fatty acid composition and gross energy. These findings suggest that 5% PAR may be a suitable practical inclusion level for juvenile black carp diets under the present experimental conditions. Before higher inclusion levels can be recommended, amino acid balancing, especially methionine supplementation or broader essential amino acid correction, improved processing to reduce indigestible fractions, and direct measurements of dietary gross energy, fatty acid profile, and chitin content are needed.

## Figures and Tables

**Table 1:** Formulations of the experimental diets.

Ingredients (%)	Diet 1	Diet 2	Diet 3	Diet 4	Diet 5
Fishmeal	20.00	15.00	10.00	5.00	0.00
*Periplaneta americana* residue	0.00	5.00	10.00	15.00	20.00
Soybean meal	25.00	25.00	25.00	25.00	25.00
Wheat flour	18.65	18.45	18.35	18.25	18.25
Rapeseed meal	10.00	10.00	10.00	10.00	10.00
Cottonseed meal	10.00	10.00	10.20	10.30	10.40
Wheat middling	8.00	9.00	9.80	10.60	11.40
Fish oil	2.00	1.60	1.15	0.75	0.30
Soybean oil	2.00	1.60	1.15	0.75	0.30
Soybean lecithin	1.00	1.00	1.00	1.00	1.00
Vitamin premix ^1^	1.00	1.00	1.00	1.00	1.00
Mineral premix ^2^	1.00	1.00	1.00	1.00	1.00
Ascorbic acid	0.05	0.05	0.05	0.05	0.05
Choline chloride	0.25	0.25	0.25	0.25	0.25
Antioxidant	0.05	0.05	0.05	0.05	0.05
Ca(H_2_PO_4_)_2_	1.00	1.00	1.00	1.00	1.00
Total	100.00	100.00	100.00	100.00	100.00

^1^ Vitamin premix (per kg diet): vitamin A, 62500 IU; vitamin D3, 15000 IU; vitamin E, 1.75 g; vitamin K3, 35.4 mg; vitamin B1, 100 mg; vitamin B2, 150 mg; vitamin B6, 150 mg; vitamin B12, 0.2 mg; biotin, 4 mg; D-calcium pantothenate, 250 mg; folic acid, 25 mg; nicotinamide, 300 mg; vitamin C, 700 mg. ^2^ Mineral premix (per kg diet): FeSO_4_.H_2_O, 200 mg; CuSO_4_.5H_2_O, 96 mg; ZnSO_4_.H_2_O, 360 mg; MnSO_4_.H_2_O, 120 mg; MgSO_4_.H_2_O, 240 mg; KH_2_PO_4_, 4.2 g; NaH_2_PO_4_, 0.5 g; KI, 5.4 mg; CoC_l2_.6H_2_O, 2.1 mg; Na_2_SeO_3_, 3 mg. Diet 1, Diet 2, Diet 3, Diet 4, and Diet 5 represent diets containing 0%, 5%, 10%, 15%, and 20% PAR, respectively.

**Table 2:** Proximate composition and amino acid content of fishmeal (FM), *Periplaneta americana* residue (PAR), and experimental diets.

Items	FM	PAR	Diet 1	Diet 2	Diet 3	Diet 4	Diet 5
Proximate composition (% dry matter)							
Crude protein	68.95	58.61	40.72	40.31	40.15	41.06	41.32
Crude lipid	7.00	23.37	12.88	12.54	12.82	12.44	13.08
Ash	14.86	5.30	6.76	6.71	7.08	6.42	6.74
Amino acid content (% dry matter)							
Essential amino acids							
Arginine	3.63	3.03	2.53	2.70	2.54	2.59	2.54
Histidine	1.97	1.62	1.00	1.07	1.16	1.22	1.26
Isoleucine	2.71	2.22	1.62	1.58	1.55	1.52	1.48
Leucine	4.64	4.04	2.73	2.68	2.64	2.59	2.56
Lysine	4.96	3.51	2.25	2.17	2.11	2.04	1.95
Methionine	1.80	0.70	0.62	0.60	0.53	0.51	0.44
Phenylalanine	2.52	2.05	1.73	1.74	1.72	1.69	1.65
Threonine	2.59	2.41	1.43	1.40	1.38	1.37	1.34
Valine	3.17	3.54	1.88	1.89	1.91	1.94	1.95
∑EAA	27.99	23.12	15.79	15.83	15.54	15.47	15.17
Non-essential amino acids							
Aspartic acid	5.72	5.17	3.54	3.51	3.45	3.41	3.37
Serine	2.15	2.22	1.38	1.32	1.36	1.38	1.32
Glutamic acid	8.12	6.80	6.52	6.40	6.35	6.31	6.21
Glycine	3.89	4.10	1.85	1.84	1.85	1.88	1.88
Alanine	4.00	4.41	1.89	1.90	1.94	1.98	2.00
Cystine	0.02	0.07	0.01	0.02	0.02	0.02	0.02
Tyrosine	1.98	4.27	0.95	1.18	1.09	1.35	1.33
Proline	2.87	3.02	2.11	2.13	2.13	2.15	2.13
∑NEAA	28.75	30.06	18.25	18.30	18.19	18.48	18.26
∑TAA	56.74	53.18	34.04	34.13	33.73	33.95	33.43
∑EAA/TAA (%)	49.33	43.47	46.39	46.38	46.07	45.57	45.38

Note: The diets were formulated to be isonitrogenous and isolipidic based on proximate composition. However, dietary gross energy, fatty acid composition, and chitin content were not directly determined in the present study. Therefore, the effects of high PAR inclusion should be interpreted as the combined effects of overall ingredient substitution, amino acid balance, lipid-source alteration, possible essential fatty acid changes, and chitin/fiber-like fractions, rather than being attributed to chitin alone. ∑EAA: total essential amino acids; ∑NEAA: total non-essential amino acids; ∑TAA: total amino acids. Diet 1, Diet 2, Diet 3, Diet 4, and Diet 5 represent diets containing 0%, 5%, 10%, 15%, and 20% PAR, respectively.

**Table 3:** Survival rates and growth performance of juvenile *Mylopharyngodon piceus*.

Items	Diet 1	Diet 2	Diet 3	Diet 4	Diet 5
Survival rates (%)	100.00 ± 0.00	91.67 ± 8.33	93.33 ± 4.41	100.00 ± 0.00	98.33 ± 1.67
Initial body weight (g)	17.18 ± 0.03	17.19 ± 0.02	17.18 ± 0.03	17.21 ± 0.02	17.20 ± 0.02
Final body weight (g)	43.30 ± 0.92	43.78 ± 1.63	42.45 ± 3.28	39.86 ± 2.00	38.04 ± 3.34
Weight gain rate (%)	152.03 ± 4.99	154.60 ± 9.22	147.10 ± 19.36	131.60 ± 19.91	121.10 ± 19.58
Specific growth rate (%/d)	1.47 ± 0.03	1.48 ± 0.06	1.43 ± 0.12	1.33 ± 0.08	1.25 ± 0.14
Feed conversion ratio	1.46 ± 0.01	1.45 ± 0.01	1.61 ± 0.16	1.67 ± 0.10	1.80 ±0.26
Daily feeding rate (%/day)	2.00 ± 0.03 ^ab^	1.92 ± 0.04 ^a^	2.04 ± 0.03 ^b^	2.08 ± 0.02 ^b^	2.02 ± 0.03 ^ab^
Condition factor (%)	1.87 ± 0.03 ^ab^	1.89 ± 0.05 ^ab^	1.94 ± 0.02 ^a^	1.93 ± 0.02 ^ab^	1.83 ± 0.02 ^b^
Viscerosomatic index (%)	9.24 ± 0.20 ^a^	8.37 ± 0.42 ^a^	9.40 ± 1.05 ^a^	9.65 ± 1.76 ^a^	14.87 ± 0.88 ^b^
Hepatosomatic index (%)	1.47 ± 0.05 ^a^	1.55 ± 0.06 ^ab^	1.68 ± 0.22 ^ab^	1.50 ± 0.34 ^a^	2.14 ± 0.08 ^b^

Data are presented as mean ± SE (*n* = 3). Values within the same row with different letters indicate significant differences according to Duncan’s multiple range test (*p* < 0.05). Diet 1, Diet 2, Diet 3, Diet 4, and Diet 5 represent diets containing 0%, 5%, 10%, 15%, and 20% PAR, respectively.

**Table 4:** Proximate composition and amino acid content of juvenile *Mylopharyngodon piceus*.

Items	Diet 1	Diet 2	Diet 3	Diet 4	Diet 5
Proximate composition (% dry matter)					
Crude protein	64.48 ± 1.35 ^a^	64.50 ± 1.38 ^a^	59.15 ± 2.09 ^ab^	53.39 ± 1.44 ^b^	56.43 ± 2.42 ^b^
Crude lipid	28.77 ± 0.28	29.99 ± 0.97	31.77 ± 1.49	30.36 ± 0.92	29.41 ± 0.58
Ash	9.65 ± 0.23	8.90 ± 0.33	8.87 ± 0.41	8.98 ± 0.39	9.80 ± 0.24
Amino acid content (% dry matter)					
Essential amino acids					
Arginine	3.31 ± 0.01	3.38 ± 0.03	3.37 ± 0.06	3.39 ± 0.03	3.39 ± 0.04
Histidine	1.54 ± 0.00	1.57 ± 0.03	1.57 ± 0.04	1.63 ± 0.02	1.61 ± 0.03
Isoleucine	2.36 ± 0.01	2.41 ± 0.03	2.39 ± 0.06	2.44 ± 0.06	2.39 ± 0.03
Leucine	4.00 ± 0.01	4.07 ± 0.04	4.07 ± 0.07	4.12 ± 0.08	4.07 ± 0.04
Lysine	4.46 ± 0.03	4.57 ± 0.10	4.51 ± 0.15	4.51 ± 0.11	4.46 ± 0.11
Methionine	1.61 ± 0.01 ^a^	1.61 ± 0.02 ^a^	1.54 ± 0.04 ^ab^	1.52 ± 0.03 ^ab^	1.45 ± 0.03 ^b^
Phenylalanine	2.25 ± 0.02	2.27 ± 0.02	2.25 ± 0.03	2.25 ± 0.04	2.24 ± 0.02
Threonine	2.23 ± 0.02	2.25 ± 0.03	2.27 ± 0.05	2.34 ± 0.03	2.34 ± 0.02
Valine	2.63 ± 0.02	2.68 ± 0.04	2.70 ± 0.07	2.74 ± 0.05	2.68 ± 0.04
∑EAA	24.39 ± 0.10	24.81 ± 0.27	24.62 ± 0.46	24.93 ± 0.41	24.61 ± 0.29
Non-essential amino acids					
Alanine	3.58 ± 0.02	3.60 ± 0.04	3.62 ± 0.07	3.63 ± 0.04	3.58 ± 0.03
Aspartic acid	5.25 ± 0.03	5.35 ± 0.07	5.32 ± 0.13	5.41 ± 0.09	5.40 ± 0.13
Cystine	0.04 ± 0.02	0.04 ± 0.01	0.04 ± 0.04	0.06 ± 0.03	0.05 ± 0.02
Glutamic acid	7.96 ± 0.07	8.04 ± 0.11	8.00 ± 0.15	8.05 ± 0.11	7.97 ± 0.20
Glycine	4.07 ± 0.04 ^a^	4.09 ± 0.09 ^a^	4.15 ± 0.10 ^ab^	4.17 ± 0.05 ^ab^	4.26 ± 0.05 ^b^
Proline	2.84 ± 0.03	2.83 ± 0.00	2.77 ± 0.06	2.82 ± 0.03	2.78 ± 0.04
Serine	2.09 ± 0.02	2.10 ± 0.03	2.07 ± 0.08	2.10 ± 0.04	2.17 ± 0.02
Tyrosine	1.78 ± 0.01	1.81 ± 0.02	1.81 ± 0.02	1.82 ± 0.04	1.80 ± 0.02
∑NEAA	27.60 ± 0.12	27.86 ± 0.31	27.79 ± 0.48	28.08 ± 0.38	28.01 ± 0.36
∑TAA	51.99 ± 0.21	52.67 ± 0.58	52.41 ± 0.95	53.01 ± 0.78	52.62 ± 0.65
∑EAA/TAA (%)	46.92 ± 0.01 ^ab^	47.10 ± 0.00 ^c^	46.97 ± 0.04 ^bc^	47.02 ± 0.09 ^bc^	46.77 ± 0.07 ^a^

Data are presented as mean ± SE (*n* = 3). Values within the same row with different letters indicate significant differences according to Duncan’s multiple range test (*p* < 0.05). ∑EAA: total essential amino acids; ∑NEAA: total non-essential amino acids; ∑TAA: total amino acids. Diet 1, Diet 2, Diet 3, Diet 4, and Diet 5 represent diets containing 0%, 5%, 10%, 15%, and 20% PAR, respectively.

**Table 5:** Metabolism indices of liver and serum of juvenile *Mylopharyngodon piceus*.

Items	Diet 1	Diet 2	Diet 3	Diet 4	Diet 5
Liver					
ALT (U/g protein)	8.51 ± 0.26 ^a^	2.89 ± 1.64 ^ab^	4.62 ± 2.15 ^ab^	8.04 ± 3.09 ^a^	1.49 ± 0.68 ^b^
AST (U/g protein)	32.47 ± 6.43 ^a^	16.04 ± 7.31 ^b^	11.67 ± 4.15 ^b^	10.13 ± 0.85 ^b^	8.36 ± 2.72 ^b^
Glycogen (mmol/g tissue)	57.45 ± 7.51	55.56 ± 6.18	50.32 ± 10.20	63.88 ± 5.03	55.25 ± 2.54
Serum					
TP (g/L)	31.75 ± 3.61	27.40 ± 1.30	24.69 ± 2.0	30.23 ± 1.43	24.53 ± 1.78
BUN (mmol/L)	5.71 ± 0.84 ^c^	8.52 ± 0.66 ^bc^	13.56 ± 0.39 ^a^	9.70 ± 1.88 ^b^	9.30 ± 0.89 ^b^
AST (U/L)	8.43 ± 3.47	12.03 ± 5.09	6.37 ± 2.28	8.63 ± 1.94	9.92 ± 1.75
T-AA (μmol/mL)	42.46 ± 3.82	42.63 ± 1.82	54.03 ± 2.44	46.14 ± 6.91	46.84 ± 6.90
TG (mmol/L)	5.20 ± 0.57	6.80 ± 0.73	5.17 ± 0.74	4.91 ± 0.50	4.94 ± 0.75
T-CHO (mmol/L)	3.44 ± 0.30	3.79 ± 0.33	3.54 ± 0.44	3.86 ± 0.58	3.93 ± 0.65
Glucose (mmol/L)	5.04 ± 0.47 ^ab^	4.20 ± 0.13 ^b^	6.80 ± 0.42 ^a^	6.68 ± 1.26 ^a^	6.10 ± 0.42 ^ab^

Data are presented as mean ± SE (*n* = 3). Values within the same row with different letters indicate significant differences according to Duncan’s multiple range test (*p* < 0.05). TP: total protein; T-AA: total free amino acids; AST: aspartate aminotransferase; ALT: alanine aminotransferase; T-CHO: total cholesterol; TG: triglyceride. Diet 1, Diet 2, Diet 3, Diet 4, and Diet 5 represent diets containing 0%, 5%, 10%, 15%, and 20% PAR, respectively.

**Table 6:** Antioxidant capacity and immunity indices in liver and serum of juvenile *Mylopharyngodon piceus*.

Items	Diet 1	Diet 2	Diet 3	Diet 4	Diet 5
Liver					
CAT (U/g protein)	8.71 ± 0.33 ^b^	10.57 ± 0.27 ^a^	6.08 ± 0.08 ^d^	7.48 ± 0.49 ^c^	6.72 ± 0.06 ^cd^
T-AOC (U/g protein)	0.91 ± 0.38	1.32 ± 0.29	0.47 ± 0.17	0.76 ± 0.30	0.61 ± 0.18
GSH-Px (U/mg protein)	96.97 ± 13.17 ^ab^	124.23 ± 13.26 ^a^	77.17 ± 2.02 ^b^	110.67 ± 5.14 ^a^	69.38 ± 8.43 ^b^
AKP (U/g protein)	0.05 ± 0.01 ^a^	0.05 ± 0.01 ^a^	0.02 ± 0.00 ^b^	0.03 ± 0.01 ^b^	0.02 ± 0.00 ^b^
ACP (U/g protein)	0.21 ± 0.01 ^a^	0.22 ± 0.02 ^a^	0.13 ± 0.01 ^b^	0.18 ± 0.03 ^ab^	0.15 ± 0.02 ^b^
LZM (U/mg protein)	3.00 ± 1.34	1.78 ± 0.72	1.01 ± 0.28	1.94 ± 0.20	1.21 ± 0.61
Serum					
CAT (U/L)	2.68 ± 1.24	4.00 ± 1.02	5.92 ± 1.11	4.12 ± 0.32	5.67 ± 0.57
T-AOC (U/mL)	4.28 ± 0.97	4.40 ± 0.21	4.19 ± 0.57	3.37 ± 0.53	3.95 ± 0.35
GSH-Px (U/mL)	1862.43 ± 196.77 ^b^	1853.97 ± 252.16 ^b^	2014.81 ± 76.31 ^b^	3098.41 ± 153.61 ^a^	1913.23 ± 37.62 ^b^
AKP (U/L)	18.03 ± 0.88	17.17 ± 0.18	16.69 ± 2.56	13.79 ± 2.50	12.32 ± 1.58
ACP (U/L)	17.22 ± 1.05	12.10 ± 3.99	16.46 ± 2.52	12.27 ± 0.81	16.26 ± 2.96
Complement C3 (U/mL)	801.68 ± 182.77	580.52 ± 164.43	644.70 ± 113.86	763.47 ± 130.46	420.19 ± 74.21
IgM (μg/mL)	448.81 ± 137.31	509.99 ± 180.20	497.85 ± 90.83	502.29 ± 78.11	305.70 ± 145.84

Data are presented as mean ± SE (*n* = 3). Values within the same row with different letters indicate significant differences according to Duncan’s multiple range test (*p* < 0.05). CAT: catalase; T-AOC: total antioxidant capacity; GSH-Px: glutathione peroxidase; AKP: alkaline phosphatase; ACP: acid phosphatase; LZM: lysozyme; IgM: immunoglobulin M. Diet 1, Diet 2, Diet 3, Diet 4, and Diet 5 represent diets containing 0%, 5%, 10%, 15%, and 20% PAR, respectively.

## Data Availability

The data supporting the findings of this study are available from the corresponding author upon reasonable request.
